# Maternal Obesity and Neurodevelopment of the Offspring

**DOI:** 10.3390/nu17050891

**Published:** 2025-03-02

**Authors:** Anna Eleftheriades, Sevasti Koulouraki, Antonios Belegrinos, Makarios Eleftheriades, Panagiota Pervanidou

**Affiliations:** 1 Second Department of Obstetrics and Gynaecology, Aretaieion Hospital, National and Kapodistrian University of Athens, 11528 Athens, Greece; annielefth-28@hotmail.com (A.E.); sevasti.koulouraki@gmail.com (S.K.); melefth@med.uoa.gr (M.E.); 2 Unit of Developmental and Behavioral Paediatrics, First Department of Paediatrics, Agia Sophia Children’s Hospital, National and Kapodistrian University of Athens, 11527 Athens, Greece; a.belegrinos@outlook.com

**Keywords:** maternal obesity, overweight, neurodevelopment, neuropsychiatric disorders

## Abstract

Background: An increasing amount of evidence, derived from both human epidemiological studies and animal research, suggests that exposure to maternal obesity in utero is linked to adverse neurodevelopmental outcomes in the offspring. These can include attention deficit hyperactivity disorder, autism spectrum disorders, intellectual disability, and cerebral palsy. Methods: A thorough search in Medline/PubMed and Google Scholar databases was performed by two independent reviewers in order to investigate the link between the exposure to maternal obesity and neurodevelopmental outcomes in the offspring. A list of keywords, including maternal obesity, maternal overweight, maternal diet, neurodevelopment, and neuropsychiatric disorders, was used in the search algorithm. Results: The existing evidence regarding the potential mechanisms through which maternal obesity may impact offspring neurodevelopment and programming, such as inflammation, hormone dysregulation, alterations to the microbiome, and epigenetics, as well as evidence from animal studies, was summarized in this narrative review. Conclusions: Maternal obesity seems to be overall associated with various neuropsychiatric and neurodevelopmental disorders. However, more robust data from future studies are needed to establish this association, which will take into account the role of potential confounders such as genetic factors and gene–environment interactions.

## 1. Introduction

The prevalence of maternal obesity has greatly increased in recent years and is considered an epidemic health issue in most countries globally. In fact, more than one third of adult women are overweight [1]. More specifically, in the United States, the country with the highest obesity prevalence, 27% of women of reproductive age are overweight (BMI of 25–30 kg/m^2^), while 37% are obese (BMI ≥ 30 kg/m^2^) [2]. Five European countries follow in this ranking, presenting percentages of maternal obesity greater than 20%, among which the UK exceeds 25% [3]. Moreover, it has been shown that nearly half of women gain more weight during pregnancy than the upper limit recommended by the National Obstetrical Societies, independently of pre-pregnancy weight [4].

Maternal obesity is a well-known risk factor for both short-term maternal complications during pregnancy, such as gestational diabetes mellitus [5,6], preeclampsia, thromboembolism, preterm delivery, labor complications, higher cesarean section rates, and infections [4,7], and long-term maternal complications, including diabetes, hypertension, and cardiometabolic morbidity [8]. Maternal obesity and an overweight status during pregnancy have been associated with adverse long-term childhood outcomes such as cardiovascular morbidity, obesity, endocrine and immune defects, and neurodevelopmental disorders. The latter seem to present an increasing prevalence in recent years, as 15% of 2- to 8-year-old children are estimated to have a neurodevelopmental disability [9].

Neurodevelopmental disorders constitute a set of conditions that usually appear early in life due to disturbances in brain development. These disruptions can lead to alterations or delays in several areas of functioning, including social skills, communication, intellectual abilities, motor skills, emotional development, attention, and memory. The origins of neurodevelopmental disorders are multifactorial, involving a mix of genetic factors, environmental influences, and their interactions. Growing evidence suggests that complications during pregnancy can impact fetal brain development, raising the risk of neurodevelopmental disorders in children, with maternal obesity being one such factor [6].

Due to this parallel observed rise in maternal obesity in pregnancy and neurodevelopmental disorders, a need to study a possible causal link between them has emerged. Correspondingly, there has been growing attention given to maternal weight status, including both the pre-pregnancy weight and excess gestational weight gain (GWG), and its impact on offspring neurodevelopmental outcomes [10]. It is already known that in utero exposure to stress and toxins can affect fetal brain development adversely. As maternal obesity creates an inflammatory uterine environment, it may be hypothesized that this unfavorable environment could induce epigenetic changes in neural tissue function, thus leading to abnormal fetal programming with long-term implications on offspring neurodevelopment [11,12].

The magnitude of the overall effect of pre-pregnancy overweight or obesity on child neurodevelopment is unknown. During the last decade, there has been growing literature on both the possible pathophysiology mechanisms and the different clinical manifestations of neurodevelopmental morbidity [13,14]. Epidemiological and animal studies have examined the correlation between maternal obesity and offspring neurobehavioral defects, including cognitive impairment/intellectual disability, attention deficit–hyperactivity disorder (ADHD), autism spectrum disorder (ASD), anxiety and depression, schizophrenia, epilepsy, and cerebral palsy (CP) [15]. Due to the steadily rising prevalence of maternal obesity globally, it seems crucial to ascertain its causative link to these neurodevelopmental disorders, so that more interventions, preventive measures, and therapies can be implemented in daily medical practice in order to improve the neurodevelopmental outcomes of children exposed to maternal obesity in pregnancy [13].

## 2. Materials and Methods

In order to identify the impact of prenatal exposure to maternal obesity on neurodevelopment, the authors conducted an extensive Medline/PubMed and Google Scholar database search of the literature in English until September 2024. A list of keywords, including maternal obesity, maternal overweight, maternal diet, neurodevelopment, and psychiatric disorders, was used in the search algorithm. All types of research papers were eligible for our narrative review. Reports were excluded if the full text was not accessible or if the paper was published in a language other than English, and no other exclusion criteria were applied. The reference lists from all the reviewed papers were screened for potentially eligible papers. The bibliography screening (title, abstract, and full text) was conducted independently by two different authors to avoid a potential source of bias.

## 3. Results

### 3.1. Pathophysiology

The mechanisms through which maternal obesity is associated with variable neurodevelopmental disorders are not yet fully elucidated. Taking into consideration the inflammatory nature of obesity, research has focused on the maternal cytokine profile, fatty acids, and oxidative stress in the fetoplacental unit, and their impact on brain development and function [16].

Oxidative stress during pregnancy is associated with increased circulating cytokines in the maternal circulation, including interleukin 1 (IL-1), interleukin 6 (IL-6), tumor necrosis factor (TNF-a), monocyte chemoattractant protein 1, and *C*-reactive protein (CRP) [17,18]. These cytokines seem to be involved in neurite growth and differentiation. Increased levels of IL-6 have been associated with the impaired innervation of the hypothalamus [19], while increased TNF-a expression is correlated with reduced neurite growth within the sympathetic nervous system [20]. Furthermore, maternal obesity has been found to contribute to an imbalance in transforming growth factor β (TGF-β), which, in turn, is associated with impaired neurodevelopment and signaling [21]. More specifically, low levels of TGF-β affect the function of microglial cells along with the differentiation, maintenance, axon growth, and regulation of synaptic electrophysiology, and they are associated with ASD [22,23,24]. On the other hand, increased levels of TGF-β have been observed in the circulating monocytes of untreated schizophrenia patients [25]. The redundant adiposity also induces lipolysis augmentation that, in turn, leads to free fatty acids (FFAs) and the aforementioned cytokine release, while maternal hepatic very-low-density lipoprotein (VLDL) secretion is increased. The placenta is known to allow the passive flow of circulating FFAs and IL-1 [10,26,27,28] to the fetus and to secrete these inflammatory cytokines towards the intrauterine environment [18,29]. Saturated FFAs also induce the release of pro-inflammatory cytokines in placental trophoblast cells [30]. These inflammatory processes activate the production of reactive oxygen free radicals (O_2−_, OH, H_2_O_2_) [31] and dysregulate the placental and intrauterine environment through increased oxidative stress, lipotoxicity, inflammation, and the over-activation of placental macrophages [18,29]. This unfavorable in utero inflammatory environment is associated with altered cytokine production and neuronal dysfunction in the fetus [32,33]. Additionally, the placentas of obese mothers present an affected vasculature and increased activated decidual immune cells, including activated decidual natural killer (NK) cells, that promote inflammatory reactions against the invading trophoblast, probably triggering hypoxia [34,35,36,37]. Further to the above, obesity is associated with chronic villitis due to increased placental infiltration by both CD^3+^ T cells and CD^68+^ macrophages, which, in turn, is correlated with cerebral palsy and neonatal encephalopathy [38]. Still, human studies have failed to find a correlation between maternal and fetal saturated fatty acid levels [39,40]. Interestingly, the maternal dietary adjustments of polyunsaturated fatty acids (PUFAs) contribute to the lipid profile of the offspring’s developing brain without affecting the systematic circulation levels of fatty acids [41]. An imbalanced diet containing high levels of *n*-6 PUFAs (from meat) and low *n*-3 PUFAs (from fish) seems to impair microglial motility and lipid composition and exacerbate the expression of pro-inflammatory genes, possibly contributing to changes in the fetal brain associated with behavioral defects [42,43]. Human studies of maternal *n*-3 PUFA supplementation showed controversial results regarding the beneficial effects on the offspring’s cognitive outcome [44,45]. Further investigation is needed to provide new leads for intervention.

Another field of research is the association of maternal adiposity with increased leptin production, hyperinsulinemia, and hyperglycemia, even in the absence of diabetes. Due to the consequential fetal hyperglycemia, insulin, glucose, and leptin signaling in the fetal brain are dysregulated [46]. Specifically, insulin secretion is increased by the fetal pancreas, while the consecutive inflammation in fetal skeletal muscles and adipose tissue lead to increased insulin resistance, thus provoking a decrease in the expression of insulin receptors and glucose transporters in the fetal brain [46,47,48], and especially in the signaling pathways of learning and memory in the hippocampus and cortex [49]. These metabolic disruptions induced by maternal obesity might also damage microglial metabolic plasticity in the offspring, which would, in turn, impair neurodevelopment [50,51]. Leptin dysfunctional signaling in the fetal brain is also considered to be another mechanism of neurodevelopmental deficits in the offspring of obese mothers. Leptin is considered an important neurotrophic factor [52], playing a critical role in stem cell growth and synaptic plasticity in the hippocampus, amygdala, cortex, and thalamus—brain regions that are involved in learning and behavioral adjustments [17,53]. Leptin has been found to regulate the microglial activation state in the rat hypothalamus and to activate cytokines-induced inflammation in vitro, impairing microglial phagocytosis and leading to a decrease in hypothalamic neuron development [54,55].

Additionally, at the base of fetal brain inflammation and oxidative stress, the signaling pathways of serotonin (5-HT) and dopamine are dysregulated, thus impairing the reward circuitry. Dysfunctional serotonin signaling in the prefrontal cortex [46], the inflammation-induced breakdown of the serotonin precursor, and reduced serotonin synthesis are some of the proposed mechanisms that increase the incidence of depression, anxiety, ADHD, ASD, and eating disorders in the offspring of obese mothers [10,46]. Similarly, impaired dopaminergic pathways are associated with schizophrenia, ASD, ADHD, and eating disorders later in life [10,56]. Finally, brain-derived neurotrophic factor (BDNF), which plays a crucial role in synaptic plasticity, neuronal differentiation, and survival, has been found to be silenced in the cortex and hippocampus [57,58] impairing the spatial learning capacity and memory in the adolescent offspring of obese mothers [59,60].

Several human studies have also identified structural changes in the brains of offspring exposed in utero to maternal obesity, with a decrease in the white matter integrity [61,62] throughout the neonatal to early-adult life. Moreover, synaptic dysfunction is observed in the brain regions involved in cognition, motor control, and sensory ability at two weeks of life [63,64]. Furthermore, the microglia’s developmental actions are impaired by maternal-obesity-induced immune activation. Thus, microglial proliferation and phagocytosis are increased, along with intensified neural progenitor cell phagocytosis and proliferative signaling, imbalanced interneuron migration, the defasciculation of the corpus callosum neural axons, the reduced extension of dopaminergic axons, apoptosis in postnatal cerebellar neurons, and increased angiogenesis in retinal endothelial cells [16].

Also, the detrimental effects of obesity are now being examined through the perspective of epigenetics. DNA hypomethylation, the differential expression of microRNA, and histone modifications have been shown to take place, impairing the microglia’s epigenetic landscape in the offspring of obese mothers [65]. This seems to be inheritable through the next generations [66,67]. These transcriptomic changes seem to be transient, as adult microglia show only mild differences in gene expression [56,68]. Maternal obesity has also been linked to deregulated oxytocin receptor messenger RNA (mRNA) levels and altered GABAergic (gamma-aminobutyric acid) signaling in the hippocampus of male offspring, which is likely to impair hippocampal development, neurogenesis, and the later-life function of social behavior [69,70,71]. Different developmental vulnerabilities might exist between the sexes, with male offspring being more susceptible to maternal-obesity-induced changes in brain gene expression [31] and chromatin-remodeling processes in the hippocampus, amygdala, and prefrontal cortex [72,73].

Moreover, the altered composition of the maternal microbiome induces increased endotoxin production, with the endotoxins traveling through the maternal circulation [74] and contributing to inflammation. This seems to be correlated with a reduction in *L. reuteri* in the fetal microbiome, which, in turn, leads to reduced oxytocin production [75] andcould thus cause behavioral deficits, ASD, and ADHD [76,77]. A maternal high-fat diet also seems to induce neuroinflammatory responses in utero that disturb iron homeostasis and increase the ferroportin and hepcidin levels. These alterations may affect processes of myelination that are probably involved in behavioral defects later in life, especially in male offspring [78].

The “dysbiosis” of the maternal gut microbiome, caused by factors such as a high-fat diet, obesity, and antibiotic use during pregnancy, can influence fetal brain development through various mechanisms. This may contribute to abnormal brain function and behavior in offspring. Fetuses do not develop in a sterile environment, and the placental microbiome of obese women before pregnancy has less diversity and abundance compared to women with a normal pre-pregnancy weight. The microbial richness decreases from the maternal side to the fetal side of the placenta [79]. Research suggests that maternal obesity can alter the uterine microbiome, particularly reducing *Lactobacilli* species, which may negatively impact the neurodevelopment of the offspring. In obese pregnancies, an imbalance in the microbiome occurs, characterized by a decline in *Lactobacilli* and an increase in pro-inflammatory microbes. This dysbiosis can result in elevated intrauterine and placental inflammation, which affect the fetal immune environment. The lipid metabolic transporters of the maternal–fetal–placental unit are also dysregulated in obesity. Consequently, the transport of essential long-chain PUFAs for fetal brain development is disturbed. The gut microbiome also produces neurotransmitters like serotonin, GABA, and other metabolites, which are transferred to the fetal brain and influence fetal neuronal differentiation. Studies in mice have shown that the microbiome can influence the serotonergic system, prefrontal cortical myelination, and the function of the hippocampus and amygdala. Structural and functional changes in the amygdala are linked to neuropsychiatric and developmental disorders, including anxiety and ASD. Understanding how the maternal microbiota interacts with fetal brain development is crucial, as it could provide valuable insights into the early-life causes of neurodevelopmental disorders [79,80].

Finally, maternal obesity affects the neurodevelopment of the offspring even postnatally, through lactation. The breastmilk of obese mothers presents an altered composition of high IL-6 that is associated with cognitive and behavioral impairment in offspring [81], as well as low neuroprotective factors such as docosahexaenoic acid (DHA), lutein, and zeaxanthin, which normally protect the offspring’s neuronal cells and retina [82,83,84].

Excessive gestational gain weight has also been associated with an inflammatory in utero environment [85]. The difference with obesity relies on the already-preexisting inflammatory factors to which the fetus is exposed earlier in pregnancy. Still, the child sex differences are related to the different adaptations of the placentas, as a female fetus’s placenta can enhance the fetal immune response to maternal obesity through gene expression adaptations, burdening fetal growth, while a male fetus’s placenta promotes intrauterine growth instead of protecting the embryo from maternal-obesity-induced intrauterine inflammation. This mechanism could explain the increased prevalence of ASD among the male offspring of obese mothers compared to the female group [86,87].

**Figure 1:**
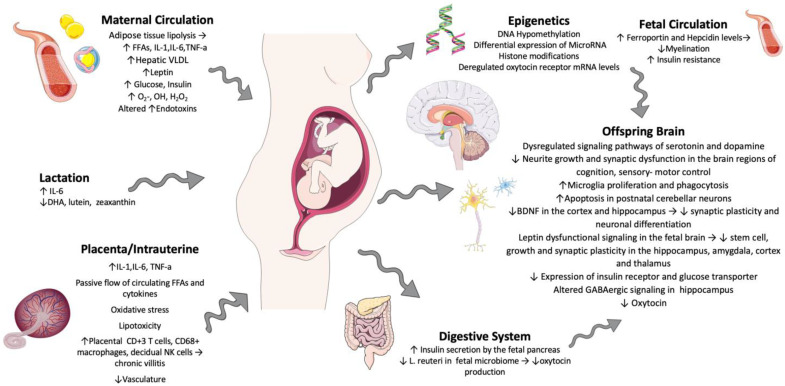
Proposed mechanisms through which maternal obesity predisposes to neurodevelopmental malprogramming; ↑: increase, ↓: decrease, →: can lead to/ is associated with.

To summarize, the intrauterine inflammatory environment that is created through maternal-obesity-induced inflammation induces detrimental changes in the fetoplacental unit through cytokine circulation, epigenetic alterations, hormone deregulation, neuronal development deterioration, and alterations to the offspring’s microbiome, along with the continuous impact of maternal obesity on the neonate through lactation. These are some of the mechanisms that have attracted attention for further research and investigation. A schematic representation is shown in Figure 1. 

### 3.2. Animal Studies

Animal studies on the impact of obesity on offspring neurodevelopment aim to complement human epidemiological observations, providing insight into the possible pathophysiological mechanisms of these adverse outcomes in addition to eliminating various environmental confounding factors, such as the postnatal environment, the socioeconomic status, and genetic predisposition [87]. Animal models of maternal diet-induced obesity are most commonly used. The most frequent type of diet consists of 30–78% fat, the so called high-fat diet (HFD), while other types of diets used include implementing both high-fat and high-carb foods to induce obesity [88,89]. Also, in the context of shared pathophysiological mechanisms between maternal obesity and the inflammation impact on offspring, various studies have focused on maternal immune activation (MIA) animal models to examine the biological pathways of adverse neurodevelopmental results in offspring exposed to maternal obesity [16].

The data from animal studies can be grouped into three major categories: alterations to brain structure, cognitive and learning impairments, and behavioral deficits. Studies on HFD rodents have observed changes in the neonatal hippocampus with impaired neurogenesis, neuronal differentiation, brain-derived neurotrophic factor (BDNF) production [89], increased lipid peroxidation [58], and microglial activation [59,90], along with changes in the serotonergic system, as reflected by the altered protein and gene expression of inflammatory pathway and serotonin system molecules [91]. Moreover, studies have shown the disturbed growth and maturation of neural stem-like cells in the cerebral cortex, ventricular regions, and hypothalamus [92,93]. Dendritic atrophy has also been found in the hippocampus and amygdala of adult rodents, disturbing neuronal connectivity [94], while the medial cortex has been shown to be poorly myelinated in male HFD offspring [78]. Finally, the male offspring of HFD mothers presented an increased blood barrier permeability [95] that induced white matter destruction and dysregulated monoamine neurotransmitter signaling.

Cognitive impairment, including hippocampal spatial learning and memory, has been observed in rodent offspring, as measured by the Morris water maze [57], novel object recognition [66,89], Barnes maze [96] T-maze [97], and Y-maze [97] tasks. Interestingly, it has been demonstrated that in utero exposure to maternal obesity has been linked to the transgenerational impairment of cognition, learning, memory, and synaptic plasticity [18,66].

Finally, behavioral abnormalities have been evaluated in rodent models with neurobehavioral tests that correlate with some human behavioral deficits. The open field test, examining hyperactivity, has shown an increased impact of in utero exposure to obesity and HFD on male-only offspring, and these offspring were resistant to low-fat dietary changes during lactation. On the contrary, decreased sociability and a preference for social novelty [75], which correlate with autism, have been observed in female-only offspring; however, this was improved with maternal dietary changes during lactation [88]. Moreover, anxiety and depression have been evaluated with the open field test, the elevated plus maze [71,98], the Morris water maze [90], and the Porsolt swim test [99,100]. Anxiety seemed to be equally noted in rodent offspring of both sexes, but was only improved in females after maternal dietary interventions. Interestingly, in a nonhuman primate model, only female offspring showed increased anxiety at four months [101]; increased anxiety was then present in both sexes by 11 months. This early-life sexual disparity is in accordance with human studies [52]. A transgenerational increase in anxiety has also been observed among female descendants [67]. Altered GABAergic and neurotrophin systems and decreased serotonergic signaling [102] in early life are considered to be the primary pathophysiological mechanisms of maternal-HFD-induced anxiety behaviors [71]. Finally, behavioral changes related to the increased intake of food rich in fat and carbs later in life [102] have been associated with an impaired mesolimbic reward pathway [103] that is based on epigenetic changes in dopamine and opioid expression and regulation in the nucleus accumbens [104] and ventral tegmental area (VTA). The observed higher striatal dopamine levels are also correlated with schizophrenia-like symptoms, including sensorimotor gating deficits and attention impairment [105]. Finally, maternal HFD during late pregnancy seems to be linked to addictive-like behaviors, such as alcohol, cocaine, and amphetamine abuse in rodent offspring [102,105].

Although animal studies have enlightened much of the pathophysiology on neurodevelopmental impairments induced by maternal obesity, they also present limitations concerning the variability in designs and methodologies, such as the use of high-fat or high-fat/high-sugar diets, the concentrations and timing, and the behavioral testing protocols, as well as the use of viral or bacterial mimics for MIA models. Furthermore, the examined animal psychiatric phenotypes, and especially mood and social behaviors, may not safely replicate human complexity. While these studies are consistent with epidemiological studies showing that maternal obesity is associated with a higher risk of several neurodevelopmental and psychiatric disorders, standardized systematic studies are required to further expand the knowledge on the association between maternal obesity and offspring neurodevelopmental deficits [106,107].

### 3.3. Human Studies

(a) Cognitive Impairment

A connection between maternal obesity and cognitive impairment in offspring has been established. Research indicates that children born to obese mothers tend to have lower intellectual quotient (IQ) scores, ranging from 2 to 3.4 points lower, compared to children born to mothers with a normal body mass index (BMI) [108,109,110]. Higher maternal adiposity has been linked to weaker cognitive, language, and motor skills in children, whereas a good-quality diet with an increased consumption of fish has been associated with better language skills [111]. Children exposed to maternal obesity also exhibit a poorer performance in reading and mathematics assessments and achieve lower grades in school compared to children of nonobese mothers [112,113]. In fact, signs of delayed cognitive development can be observed in these children at an early age [114,115]. A recent study by Dong et al., conducted in China and published in 2023, found a significant correlation between maternal BMI, weight gain during pregnancy, and variations in neurobehavioral development in children at the age of 2. The investigators used a Chinese version of the Bayley scales [116]. Lastly, it is worth noting that, among urban, low-income, and racial minority populations, there have been reports of sex differences in the association between maternal obesity and child cognition [117,118].

(b) ADHD

The association between maternal overweight, obesity, and severe obesity (BMI ≥ 35 kg/m^2^) and the likelihood of an ADHD diagnosis in children was observed in four large Nordic birth cohorts [119,120]. Specifically, it was discovered that maternal overweight, obesity, and severe obesity were linked to a higher risk of an ADHD diagnosis, with the percentages ranging from 23% to 28%, 47% to 89%, and 88% to 95%, respectively 119–123. Additional studies that focused on ADHD symptomatology rather than clinical diagnoses also reported an elevated risk of ADHD symptoms in children whose mothers were obese [121]. Fuemeller et al. reported that a pre-pregnancy BMI ≥ 35 was positively associated with higher ADHD symptoms and worse inhibitory control and attention. In the same study, greater-than-adequate GWG was related to a worse working memory and planning [122]. A large Greek cohort examining 772 mother–child pairs also found that maternal obesity was associated with a significant score reduction in general cognitive ability, perceptual performance, quantitative ability, executive functions, increased behavioral difficulties, and ADHD symptoms at preschool age [123]. Grudzinski et al. showed that the offspring of obese mothers required significantly more physician visits and mental health care service use for conduct disorder (IRR: 1.25, 95% CI: 1.08–1.45) and ADHD (IRR: 1.45, 95% CI: 1.24–1.69) [124]. Lastly, a recent study by Dow et al. suggested that child hyperactivity-inattention symptoms appeared more frequently in the offspring of mothers with a pre-pregnancy BMI, in both at-term and preterm children at 5 years of age, which is an important finding, since prematurity constitutes an independent risk factor for neurodevelopmental disorders [125].

(c) ASD

A majority of epidemiological studies have found that pre-pregnancy obesity increases the odds ratio for ASD in offspring by 1.3–2.05-fold [126,127,128,129,130]. These include a multi-site case-control study of 1496 children published in 2019, which showed that children with autism were more likely to have mothers with a higher weight gain during pregnancy, and this risk of autism was higher if the mothers were also overweight before pregnancy [128]. Excessive GWG on its own also seems to increase the risk for ASD in offspring by 10–58% [87,131,132]. A study by Bilder et al. found a significant association between ASD and pregnancy weight gain (the adjusted odds ratio was 1.17 for each 5 pounds of weight gained), which also applied to cases with an IQ within the normal range [132]. Among obese and overweight women, excessive GWG also seemed to exacerbate the risk of ASD [126,131] as well as severe prematurity (gestational age ≤ 30 weeks) [133] and maternal pre-gestational diabetes [127].

(d) Anxiety, depression, and internalizing behaviors

Multiple large population studies have indicated a connection between the pre-pregnancy BMI of mothers and the occurrence of anxiety and depression in their children. For instance, a cohort of Swedish children revealed that maternal pre-pregnancy obesity was linked to a twofold higher risk of displaying negative emotions, such as sadness and fear, as assessed by teachers [134]. This increased risk persisted throughout adolescence. Additionally, two cohorts that followed children until the age of 17 reported an elevated likelihood of withdrawal, depression, and anxiety among children born to mothers who were either obese or overweight [134,135]. Another study conducted by Parker et al. in 2022 found that pre-pregnancy obesity was associated with higher risks of internalizing behaviors, as reported by teachers and measured using the Teacher Report Form (TRF) between the ages of 5 and 12 [136].

(e) Intellectual disability and cerebral palsy (CP)

Maternal obesity has been linked to a significantly higher risk (ranging from 52% to 73%) of intellectual disability (characterized by an IQ below 70) in their children [137]. Remarkably, this association holds true even for mothers who undergo assisted reproductive therapy, such as in vitro fertilization and intracytoplasmic injection [138,139]. In terms of the cerebral palsy (CP) risk, a few studies indicate an increased likelihood among children of obese mothers. Moreover, the severity of obesity seems to have a dose-dependent effect, with more adverse outcomes observed in children of severely obese mothers [137,140,141]. According to a meta-analysis conducted by Xiao in 2018, which included nearly eight million participants, the relative risks of CP in the offspring of overweight, obese, and severely obese mothers were found to be 1.29, 1.45, and 2.25, respectively [142]. However, it is important to note that the association varied when factors such as the study location, the design, and specific confounding variables were taken into account.

## 4. Discussion

As maternal metabolic conditions such as diabetes, obesity, and hypertension have become more common, the occurrence of neurodevelopmental disorders in children has also risen. Current evidence suggests that maternal obesity is associated with changes in the uterine environment, which can potentially result in the unfavorable programming of the developing fetal brain, therefore predisposing children to neurodevelopmental disorders. Large studies tracking maternal–child cohorts have found an association between maternal metabolic diseases and neurodevelopmental and psychiatric disorders in children, including a higher risk of ASD, ADHD, intellectual disability, anxiety, and depression. These associations show, however, small to moderate effect sizes. On the other hand, animal studies have provided valuable insights into the mechanisms that might explain how maternal diabetes and metabolic conditions affect the developing fetal brain. The potential mechanisms for these adverse effects include oxidative stress; inflammation; the dysregulation of insulin, glucose, and leptin signaling; and impaired dopaminergic and serotonergic signaling.

## 5. Future Directions

Even if the data are promising, more evidence from larger and better-controlled studies that examine potential confounders is needed to support this association. The external variables that should be considered include the maternal and paternal medical history and genetic profile, maternal life stress, and social and environmental factors such as maternal education and income. Most importantly, maternal obesity is a confirmed risk factor for various obstetric complications such as gestational diabetes mellitus, gestational hypertension and preeclampsia, venous thromboembolism, congenital anomalies, and perinatal mortality [143]. So far, most studies have not accounted for these factors or for maternal psychiatric disorders, which can also influence the development of psychiatric disorders in offspring through various mechanisms. When adjusting for external factors, the reported associations were weakened in some studies [138]. For instance, establishing a definitive connection between maternal obesity and the development of anxiety and depression in children is challenging, since the offspring of obese mothers are more likely to be overweight, and this, in itself, is linked to increased rates of anxiety and depression [144,145]. As far as CP is concerned, one of the studies that investigated its potential mediators found that part of the increased risk for the neonates of obese mothers was mediated by asphyxia-related complications and not from overweight itself [146,147].

Of note, the complexity of determining a conclusive association between maternal obesity and the onset of neurodevelopmental disorders in children also arises from differences in how maternal obesity is defined or measured (e.g., BMI thresholds, gestational weight gain) and how neurodevelopmental outcomes are assessed. The included studies were characterized by heterogeneity due to differences in the diagnostic methodology of neurodevelopmental disorders; while some studies used clear diagnostic criteria, others used questionnaires or other tools to assess symptoms, and in some cases, the symptoms were parent-assessed [135]. Furthermore, the way in which maternal obesity was assessed in the studies (such as using BMI alone) might not capture all aspects of obesity, such as fat distribution or metabolic abnormalities, which could have different impacts on offspring neurodevelopment. Similarly, neurodevelopmental outcomes often span a wide spectrum of conditions, and different methods of assessment might not capture the full range of neurodevelopmental issues associated with maternal obesity.

## 6. Conclusions

In an effort to investigate the factors that dysregulate the uterine environment and predispose children to chronic disease, identifying those that are modifiable is essential in order implement effective early interventions. The occurrence of obesity during pregnancy is experiencing a swift rise, and this trend could potentially lead to health implications not only for mothers, but also for their children, even across generations. Epidemiological investigations have established a correlation overall between maternal obesity and various neuropsychiatric disorders. The high rates of obesity among young adults and women of childbearing age make the link between maternal obesity during pregnancy and impaired offspring neurodevelopment a significant public health concern and highlight the need to examine these exposures to help prevent offspring mental illness. More robust data are needed to prove the causal relationship between exposure and outcome, since the reliability and potency of the studies vary, partly due to concerns regarding their research methodology. Regardless, it is essential to recommend antenatal interventions for mothers who are obese or overweight to prevent or reverse the neurodevelopmental malprogramming resulting from an unfavorable intrauterine environment.

## Data Availability

No new data were created or analyzed in this study. Data sharing is not applicable to this article.
